# On the Use of the Blind Gut of Fowls as a Plug in Epistaxis

**Published:** 1851-09

**Authors:** 


*On the use of the Blind Gut of Fowls as a plug in Epistaxis.*—Dr. R. Hamilton, of Morristown, Belmont Co., Ohio, recommends this plug as peculiarly adapted to the treatment of Epistaxis. The following is the mode of application:

"Having freed the gut of all offensive matter, slip the cut end of it over a small quill, secure the attachment of the quill and gut by a few turns of pack-thread round the gut where it embraces the quill: pass a probe or knitting-needle through the barrel of the quill to the shut end of the gut; clear the nostril and throat of all clots, pass the gut on the probe along the floor of the nostril until it passes a little into the pharynx, taking especial care to have the concave side of the gut upward; withdraw the probe leaving the gut in the nostril. With the mouth applied to the quill blow forcibly into the gut, distending it as much as possible; tie the loose end of the gut securely between the quill and the nose, cut off the end of the gut containing the quill; and the operation is done. If medicated fluids or ice water are preferred, charge a syringe with them, introduce the point of the syringe into the quill, force its contents into the gut, and tie as above.

Another advantage the gut enjoys above every other plug is, that as soon as the coagulum has hardened so as to secure the safety of the patient, it may be punctured, and its contents allowed to escape, relieving the nostril of an annoying sense of fulness occasioned by necessary stretching of the parts. This may be done, long before the secretion of mucus from the Schneiderian membrane will have so loosened the clotted blood around the plug that it may be removed with safety; from three to four days being required to complete that process."—*Trans. of Belmont Medical Society, for* 1847.

